# Laser induced white lighting of graphene foam

**DOI:** 10.1038/srep41281

**Published:** 2017-01-23

**Authors:** Wieslaw Strek, Robert Tomala, Mikolaj Lukaszewicz, Bartlomiej Cichy, Yuriy Gerasymchuk, Pawel Gluchowski, Lukasz Marciniak, Artur Bednarkiewicz, Dariusz Hreniak

**Affiliations:** 1 Institute of Low Temperatures and Structural Research, Polish Academy of Science, 50-422 Wroclaw, Poland

## Abstract

Laser induced white light emission was observed from porous graphene foam irradiated with a focused continuous wave beam of the infrared laser diode. It was found that the intensity of the emission increases exponentially with increasing laser power density, having a saturation level at ca. 1.5 W and being characterized by stable emission conditions. It was also observed that the white light emission is spatially confined to the focal point dimensions of the illuminating laser light. Several other features of the laser induced white light emission were also discussed. It was observed that the white light emission is highly dependent on the electric field intensity, allowing one to modulate the emission intensity. The electric field intensity ca. 0.5 V/μm was able to decrease the white light intensity by half. Origins of the laser-induced white light emission along with its characteristic features were discussed in terms of avalanche multiphoton ionization, inter-valence charge transfer and possible plasma build-up processes. It is shown that the laser-induced white light emission may be well utilized in new types of white light sources.

New physical functionalities arising from two-dimensional carbon structures like graphene, discovered for the first time by Geim and Novoselov^1^, have been a subject of massive investigations over the last years. Its unique physical properties like electrical conductivity and mechanical properties position this material as an unquestionable leader for fast technological applications. Optical properties of graphene have also been investigated by a number of authors. However, as a zero band gap semiconductor, graphene is currently not seen as an interesting candidate for light emission devices^2^,^3^,^4^,^5^, as no light emission has been detected from pristine graphene. On the other hand, chemically modified derivatives like oxidized graphene (GO) are able to break this inconvenient situation, exhibiting broad band emission.

The broad band white light emission induced by irradiation of nano- and microcrystalline powders in vacuum with focused beam of infrared lasers has been reported recently. Roura and collaborators^6^,^7^,^8^ have reported in several papers the exotic, as they called, broadband photoluminescence of silicon nanoparticles irradiated in vacuum with relatively low intensity IR laser diode. They have discussed this emission in terms of radiative thermal emission. However, they have also noticed that the temperature of the emitting Si semiconductor nanoparticles was too low to emit a measurable thermal radiation resulting from multiphoton excitation. The observed photoluminescence has demonstrated a supralinear dependence on laser power that may be well explained within the quantum model of multiphoton excitation. The efficient broad band anti-Stokes white emission of dielectric rare earth doped oxides in powdered samples in vacuum upon irradiation with focused beam of infrared laser has been reported in 2010 by Wang and Tanner^9^. This emission was characterized by relatively low temperature of the emitting sample, threshold behavior of the intensity increasing exponentially with the excitation power and high energy efficiency. Since then, the white emission has been observed in rare earth doped nanocrystalline powders, ceramic, glasses and bulk crystals, as investigated by several groups^10^,^11^,^12^,^13^,^14^,^15^,^16^,^17^,^18^,^19^,^20^. The bright white emission was also reported for undoped nanocrystalline powders by the Di Bartollo group^21^,^22^,^23^.

Laser induced white light emission of graphene quantum dots has been investigated by Ryo Sekiya *et al*.^2^. The observation of sustained laser induced incandescence of carbon nanotubes in vacuum seen in the visible spectrum has been reported by several authors^24^,^25^,^26^. The results have been discussed in terms of blackbody radiation. Recent studies on light emission from graphene ceramics have shown that white-light emission may also be observed under intense laser excitation^27^. In the experiments, the graphene ceramics were compacted from multilayer (1–4 sheets each) graphene flakes by isostatic high pressure sintering at 8 GPa and 500 °C. The laser induced white emission (LIWE) was observed in vacuum conditions under the irradiation of a focused infrared laser beam. It has been observed that the LIWE process is a threshold anti-Stokes emission characterized by moderate temperatures (lower that 400 °C) of the emitting samples, being in evident conflict with the standard black body radiation model. It has also been shown that the power dependence of the LIWE intensity increased nonlinearly with excitation power density, *I* ∝ *P*^*n*^, where *n* = 5.15. Although the emission mechanisms responsible for the LIWE are currently far from being well understood, electronic nature of the process may presumably be associated with opening of the band gap in graphene due to the charge transfer states as result of multiphoton ionization. This theoretical approach seems to be well justified by the previous experimental data. In the present work we report the studies of LIWE in graphene foam. The results may find application in the design of a next generation of green white light sources.

## Experimental

### Measurement details

The graphene foam synthesis was carried out by the sol-gel method with graphene oxide used as a precursor. Detailed description of the synthesis can be found in the Supplementary Materials. Morphology of the sample was analyzed using Jeol JSM-6610LVnx scanning electron microscope (SEM). The Raman spectra were collected using Renishaw inVia Raman Microscope (20x magnification) equipped with VIS laser (514 nm, 10 mW) and CCD camera under ambient conditions. The photoluminescence (PL) emission spectra were measured using 975 nm continuous wave (CW) semiconductor laser diodes (LD) as an excitation source and the Avantes AVS-USB2000 CCD spectrometer as a detector. Low pressure conditions were achieved using the glass vacuum cell supplied with Pfeiffer turbomolecular pump unit (TMH071P). The best vacuum level reached with this setup was 10^−4 ^hPa. The PL quantum yield (QY) measurements were perfumed using a 2″ integrating sphere (ThorLabs) attached to an Ocean Optics USB2000+ CCD detector and a 8 W 975 nm LD. Photoconductivity measurements were performed at vacuum with a four-terminal sensing setup, utilizing silver electrodes and golden wiring and a Keithley 2400 Source-meter with 100 mV of probing voltage. The electron emission experiments were performed at vacuum (10^−4 ^hPa) in a typical two electrode setup. The anode-cathode distance was set to ca. 100 μm by thin glass spacers. The graphene was deposited at the surface of the cathode by sputtering of the material in the electric arc. The electron emission was measured in a wide range of the electric field intensity (0–7 V/μm).

## Results and Discussion

### Laser-induced white light emission

**Figure 1:**
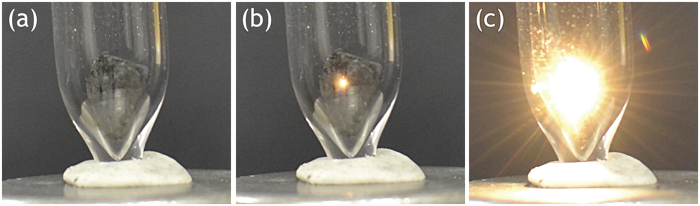
The images of (**a**) the graphene foam in a vacuum cuvette; (**b**) the photo of the graphene foam emission demonstrating lighting only from the spot at surface of graphene foam; (**c**) The photo of laser induced intense white light emission of the graphene foam.

The sample of graphene foam was placed in vacuum (Fig. 1a) and subjected to interaction with a focused beam of excitation laser with the emission wavelength of 975 nm. In the course of LIWE experiments it was found that the intense white lighting occurs only from the spot of the focused laser beam illuminating the front of the graphene foam surface (Fig. 1b). With the increase of the laser power, a very bright emission was apparently coating surface of graphene foam (Fig. 1c). It has to be pointed out that the LIWE emission is highly confined to the laser point spot. No transmitted LIWE light was observed, even for thinned (ca. 1 mm–2 mm) samples.

**Figure 2:**
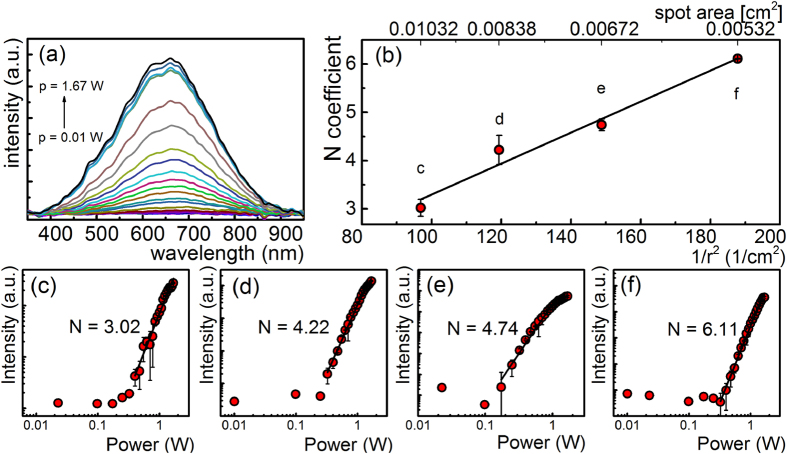
(**a**) The optical power dependence of LIWE intensity from graphene foam under 975 nm laser diode excitation. (**b**–**f**) Influence of the spot diameter on the order parameter *N* of LIWE power dependence.

The LIWE spectra were investigated as a function of excitation power with a focused beam of a CW laser (see Fig. 2).

The spectra illustrated in Fig. 2a are characterized by a broad band emission centered at ca. 650 nm with small features at the envelope resembling vibrational progressions. The exact origin of such behavior is still unclear at the present stage of the investigation, although the detector sensitivity characteristic has been excluded as a possible explanation. The observation of the white emission centered at 650 nm has already been discussed by our group for graphene ceramics in terms of a gap opening due to the light induced sp^2^ → sp^3^ phase transition^27^. However, the LIWE emission from the graphene foam is far more intensive than the one observed in the case of graphene ceramics, giving an important premise about the surface and the surface morphology impact on the emission mechanisms. It was observed that the LIWE intensity increased exponentially with increasing the excitation power. Assuming that this dependence is well scaled according to the multiphoton absorption rate the LIWE intensity may be described by the formula *I* ∝ *P*^*n*^, where *P* is the power of the incident laser beam and *n* is the order parameter usually combined with a number of absorbed photons. However, in course of experiments, we have noticed that with increasing excitation density, the order parameter *n* increases. The power dependence of LIWE was measured at different spot size of focused beam of incident laser operating at 975 nm and the results are shown in Fig. 2b–f. The LIWE intensity increased by two orders with the decrease of the spot diameter of the focused laser beam. Figure 2b shows the dependence of the order parameter *n* of the LIWE on the spot diameter. One may see that *n* increases linearly with the decrease of the spot ranging from 3.02 to 6.11. Linear character of this trend may be well correlated with the linear change of the optical power density. The spot of incident focused laser beam observed at the highest LIWE intensity was determined to be 0.05 cm^2^. It means that with the decrease of the laser fluence, the parameter *n* cannot be directly associated with the number of absorbed photons, but should be rather correlated with the electron density in avalanche ionization^28^,^29^. An additional support for this mechanism is the appearance of a power threshold that cannot be directly related with the multiphoton absorption.

The stability of LIWE intensity was measured in a long time period. The intensity remained stable and did not decrease in time (see Fig. S3). The white light was not observed from the back surface of the sample and did not spread on its surface, meaning that the light is strongly absorbed by graphene.

**Figure 3:**
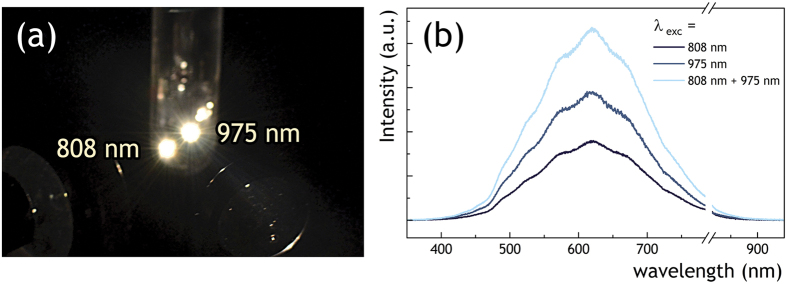
(**a**) The photo of LIWE under simultaneous illumination with two lasers operating at 808 and 975 nm of the graphene foam and (**b**) the respective LIWE spectra measured for single and double excitation at 1.6 W excitation power conditions.

To investigate the problem of additivity of the LIWE intensity with regard to its local character, a double laser beam experiment was performed. Figure 3a presents an image of graphene foam sample illuminated with two laser beams operating at 980 nm and 808 nm, observed as two separate spots. The spectra measured for the separate and summary excitations are shown in Fig. 3b. The resulting spectrum is of nearly doubled intensity. This shows that the LIWE intensity may be increased for samples excited by many focal points, opening the way for the commercial applications of the phenomena.

**Figure 4:**
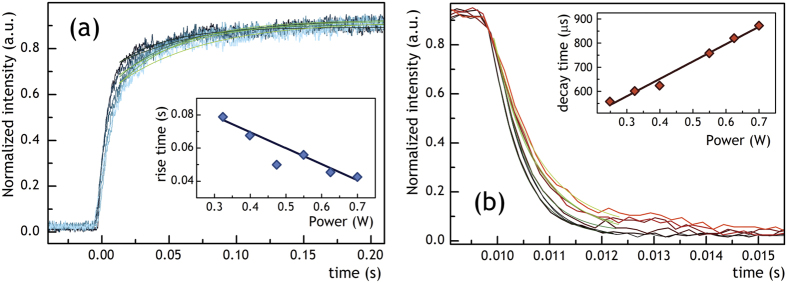
The (**a**) build-up and (**b**) decay times of LIWE of graphene foam in dependence on excitation power.

Impact of the excitation power density on the build-up and decay times of LIWE intensity after switching on and switching off of the incident laser excitation is presented in Fig. 4. The curves were analyzed using an exponential decay model. The observed build-up time decreased linearly with the excitation power from τ = 0.08 s at 0.3 W to τ = 0.04 s for 0.7 W, whereas the decay time decreased from 550 μs at 0.25 W to 890 μs at 0.7 W. It is important to note that the decay times were much shorter than the build-up times. Furthermore, the build-up and decay times were much shorter in comparison to earlier reported by our group in graphene ceramics^27^.

The temperature of LIWE graphene foam sample was measured by using nanothermometry technique^27^ with the LiLaP_4_O_12_:Er^3+^, Yb^3*+*^ nanocrystals as the thermal sensors. The nanoparticles in powdered form were deposited manually at the surface of the graphene foam. The results of the measurements can be found in the supplementary materials (see Fig. S3). The temperature of LIWE from the graphene foam sample was determined to be about 500 °C at the excitation power density of 4000 W/cm^2^, corresponding to the laser optical power of 1.5 W. The measured temperature is much lower than could be predicted using the concept of black body radiation.

### Photoconductivity of the laser irradiated graphene foam

**Figure 5:**
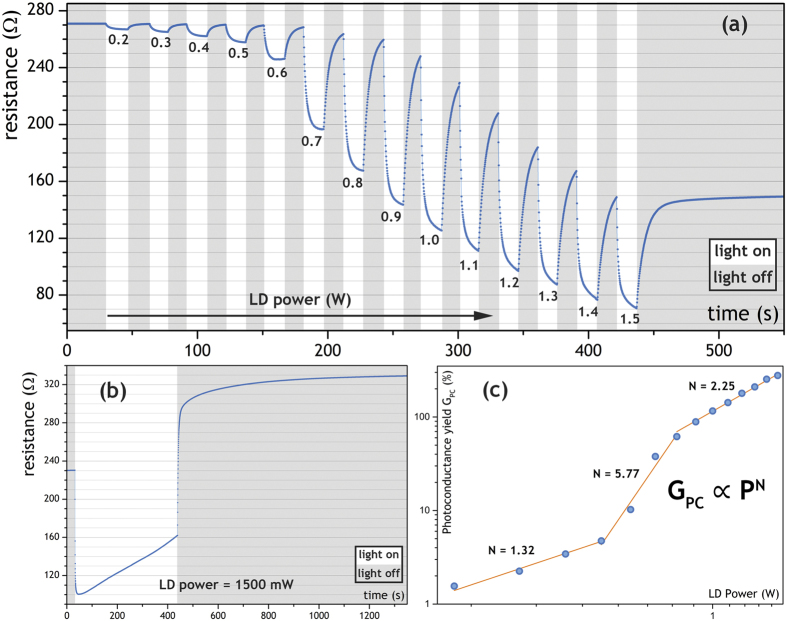
Photoconductance of the graphene foam: (**a**) change of the photoresistance in time, showing consecutive on/off cycles of 975 nm laser light source in steps of 100 mW laser diode power; (**b**) a single on/off cycle exposing effects of long-term irradiation; (**c**) LD power dependence of photoconductance yield G_PC_ in double logarithmic scale.

Photoconductivity of the graphene foam irradiated with intense infrared laser beam was carried out at the room temperature in vacuum. The graphene foam sample was measured using a four-terminal sensing setup for time-dependent resistance measurements. The results of the photoconductivity measurements obtained using an infrared 975 nm LD as the excitation source are shown in Fig. 5. The measurement was performed in on/off cycles of constant time duration of 15 seconds under a focused infrared illumination. Under the influence of the laser excitation, a significant drop in the resistivity of the sample was observed (Fig. 5a). It is also well seen that the phenomenon is of threshold nature and is strongly gaining its magnitude above the optical excitation power of ca. 0.7 W, which is well correlated with the bright white light emission occurrence. On the other hand, long-term exposure of graphene foam to a high-density infrared laser beam yields an opposite effect. After an exposure lasting several minutes, resistance of the sample grows to a value much higher than in its initial state (Fig. 5b). Furthermore, a steady decrease of sample’s conductivity during the laser beam exposure is clearly observed as a linear deviation from the exponential shape of the curve. As such, presence of two competitive effects, occurring simultaneously during high power laser excitation, should be considered. Due to the mechanical properties of the highly porous and lightweight graphene foam, as well as absorption of IR photons resulting in high ionization, it is supposed that ion sputtering takes place and that this kind of electrical degradation stems directly from partial removal of the conducting electrons. After switching-off laser irradiation, the resistance rapidly growths up from 160 Ω to 300 Ω and subsequently starts to increase very slowly in 10 min to reach the value of 330 Ω. This process is presumably associated with the cooling of the sample.

Effectiveness of these processes may be well described considering a relative factor called photoconductance (PC) yield defined as 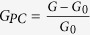, with 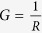 where R is the electrical resistance, G is the conductance and G_0_ is the initial (dark) value of the conductance. The G_PC_ dependence on the excitation power is shown in Fig. 5c. One may see that the G_PC_ exhibits a nearly linear dependence for small (<0.4 W) laser powers. The highest dynamics of the G_PC_ is observed between the 0.4–0.7 W with the N = 5.77, which is in good correlation with the earlier results, as well as the white-light emission threshold point. Presence of a third stage showing the attenuation of the G_PC_ rise is more difficult to rationalize, however it may be interpreted in connection to the limiting conditions defining the saturation regime. The strong exponential increase of the photoconductance yield with power is well scaled with the multiphoton absorption formula *G*_*PC*_ ∝ *P*^*N*^, where N is close to the values determined for the LIWE intensity, evidently indicating its multiphoton induced character.

### Electron emission from the graphene foam

**Figure 6:**
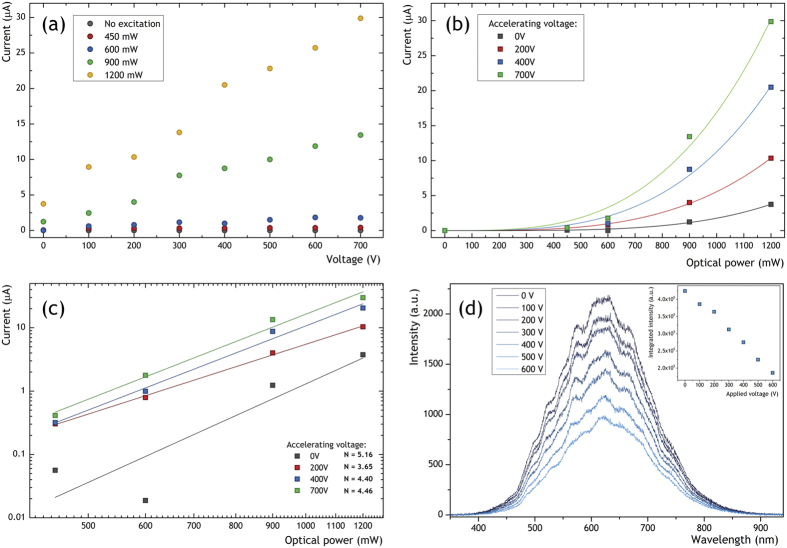
(**a**) Electron emission from the excited and non-excited graphene foam sample. (**b**,**c**) Electric current in the circuit as a function of optical excitation power (in linear and logarithmic scale, accordingly). (**d**) Intensity of the white light emission as a function of the intensity of the electric field applied between the anode-cathode setup under 400 mW of 975 nm laser diode excitation. Accelerating voltage was used as a parameter.

Some additional physical premises about the white light emission mechanisms were found measuring the electron emission effect from the non-excited and laser excited graphene (see Fig. 6). The electron emission effect was measured in a two electrode setup, with the electrode distance of ca. 100 μm and the vacuum level of 10^−4 ^hPa. No electron emission was found for the non-excited samples in the range of electric field up to 7 V/μm (Fig. 6a). Entirely different behavior was observed for the laser excited samples, showing large electron current emitted from the excited sample. It was found that the number of the emitted electrons (up to 30 μA) was highly dependent on the optical excitation power (Fig. 6a–c). Significant values of the electron current were observed even for low values of the laser excitation. Moreover, the excited samples were able to emit electrons even for 0 V applied between the anode and the cathode, giving rise to the electron current in the circuit which was clearly dependent on the laser excitation power. Presence of the electric current flow at 0 V bias conditions between the electrodes may be well understood if one take the anode-cathode setup as a flat capacitor.

Presence of a large number of electrons close to the cathode region is then responsible for induction of a positive charge at the opposite electrode, giving rise to the electronic current flow through the circuit. Such behavior clearly shows that the intense laser field is responsible for efficient electron emission from the excited samples.

Different mechanisms may be considered, including bending of the potential barrier energy at the foam-vacuum interface, photo-ionization effects and plasma build-up. Due to the foam character of the sample consisting of large number of sharp edges, the electric field may be increased to very large values by the shape factor of the sample surface, giving rise to an efficient electron emission followed by a gradual degradation of the sample’s surface. It is also likely that the thermal processes are responsible for the electron emission^30^,^31^, as the emitting samples seem to be hot enough (ca. 500 °C) to observe such process. It has to be pointed out that the process of plasma build-up at the sample-vacuum interface may explain the threshold behavior of the white-light emission as well as the multiphoton absorption processes. As it was shown that the electron emission process plays a crucial role in the LIWE, it was important to investigate the dependence of the LIWE intensity on the external electric field. The experiment was performed in a planar two-electrode setup. Each electrode was formed from a thin glass plate (ca. 1 mm) one-side covered with a 100 nm indium-tin oxide (ITO) layer used as transparent electric electrode. The graphene foam was placed between the glass slides in a form of ITO/Glass/Graphene foam/Glass/ITO. In such setup it was possible to apply a high voltage bias along with effective excitation of the sample by a laser beam. Significant diminishing of the emitted white light with the increase of the electric field intensity between the setup was observed (Fig. 6d). This suggest that the electrons’ impoverishment may be responsible for the decrease of the LIWE intensity. An empirical formula may be used to describe such process 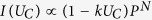, where *I* is the LIWE emission intensity, *U*_*C*_ is the applied bias voltage, *P*^*N*^ is related to the power dependent white-light emission without the electric field and *k* is the scaling parameter.

Interaction of the focused beam with the surface of graphene foam in vacuum involves a number of different processes, such as: multiphoton ionization, light emission, photocurrent generation, electron emission, thermal heating and plasma generation. In result of the multiphoton ionization, generation of free electrons occurs, inducing a change in the refraction index according to the optical Kerr effect *Δn*_*Kerr*_ = *n*_*2*_*P* that contributes to the self-focusing of incident laser beam. The Kerr refractive index *n*_*2*_ reaches a giant value for graphene close to 10^−7 ^cm^2^/W, which is almost 9 orders larger than in dielectrics^32^. Since the refractive index n_2_ is larger at the center of the focused laser beam than at the wings, it may be treated as an additional lens, drastically enhancing multiphoton ionization via avalanche.

The total photocurrent density *J* results from multiphoton ionization contributing to the electron conductivity density - *J*_*EC*_ in graphene foam, electron emission (ablation outwards) from its surface to vacuum - *J*_*EE*_ and thermionic emission density - *J*_*TE*_ associated with high temperature


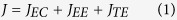


Since the multiphoton ionization rate is well scaled by power dependence *k*_*MPI*_ ∝ *P*^*N*^, also the photocurrent density should be scaled by similar relation, where *N* = *W*/*ħɷ* and *W* is called the work function related to the band gap energy between the valence and conduction bands.

The thermionic electron emission field density is described by the Richardson formula


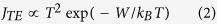


where *T* is temperature and *k*_*B*_ is Boltzmann constant. Basing on the relation (2) one may estimate that the thermionic emission for graphene foam is rather low since the temperature of LIWE is rather low about 500 °C. The total electron density generated by ionization consists of the photocurrent promoted from valence band to conduction band and electron field emission from graphene surface by ablation forming a plasma plume.

An inspection of the exponential power dependence of photoconductivity in Fig. 5 and the electron emission in Fig. 6 clearly points at the multiphoton ionization as a source of the free electrons generation. The photocurrent observed in photoconductivity measurements (Fig. 5) might be also be related to photoconductivity *J*_*EC*_ whereas the increase of resistivity during the laser excitation might be related to electron field emission *J*_*EE*_ and the thermionic emission *J*_*TE*_.

**Figure 7:**
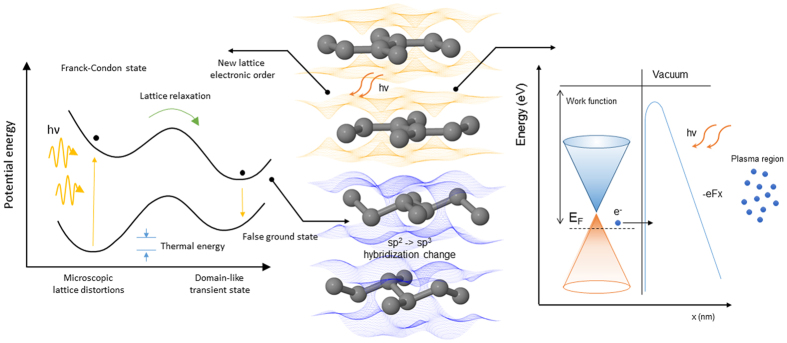
Schematic illustration of different mechanisms responsible for the white light emission from the graphene foam.

Basing on the experimental results, it is found difficult to point out just one mechanism of the LIWE emission at the current stage of understanding. One shall rather see this process as a dynamical and complex phenomena. Interaction of the focused laser beam with the surface of graphene foam placed in vacuum involves a number of different processes. They are i.e. multiphoton ionization leading to photon absorption, photocurrent generation, electron emission, thermal heating, light emission, removal of volatile adsorbents and plasma generation. Few of them which are seen to have a dominating character are illustrated in the Fig. 7.

Due to the chemical properties of carbon and its relative ease of hybridization change, it is likely that intense optical excitation used during the experiments is able to temporally disturb the electronic order of graphene’s ground state. Efficient charge transfer processes, resulted charge instability and proximity of different graphene layers may lead to strong Coulombic interactions and formation of the sp^2^ → sp^3^-like hybridization change. It is important to note that such charge transfer may occur not only by the excited state of the carbon atom, but also via the inter-valence charge transfer mechanisms leading to a number of possible and effective pathways of charge migration. Similar instabilities in graphene have already been discussed in the literature, giving rise to so called diaphite i.e. highly interacting graphene bi-layer^33^. Numerical calculations of such transient structures have shown that a small band-gap may be opened in the two layer system, which may be responsible for radiative relaxation in graphene that has already been discussed in our previous work^27^.

Although such mechanisms are convincing and the diaphite structures have already been observed in the literature, it is still difficult at this point to match the theoretical results with the experimental data. This phenomena needs more time to be better recognized and investigated.

### Potential application of LIWE of graphene foam for light bulbs

**Figure 8:**
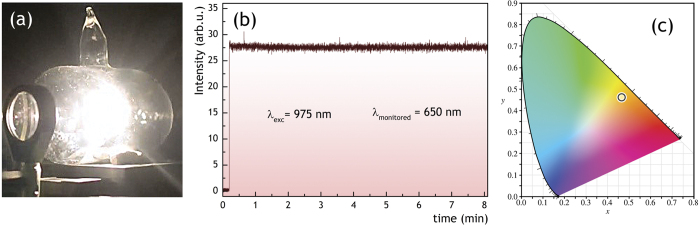
(**a**) The photo of white emission from light bulb with graphene foam. (**b**) The stability of the emission intensity of the graphene foam under 975 nm laser diode excitation. (**c**) the chromatic coordinates (CIE 1938).

The LIWE light bulb shown in Fig. 8a was made from a sealed glass container in a form of a tube with diameter of 5 cm and length of 10 cm. Graphene foam was placed in a basket of thin copper wire and hanged inside in lowered pressure atmosphere (0.1 mbar). The graphene foam was irradiated with focused beam of 980 nm laser diode. Intense brightness of white lighting was obtained at irradiation power of 1.5 W.

The stability of LIWE intensity of graphene foam was measured at emission peak at 650 nm for a long time period (8 min) (Fig. 8b) under very high optical density excitation conditions. No decrease in intensity was observed for the whole duration of the experiment, suggesting the graphene as a suitable candidate material for stable white light sources. The luminous efficiency of LIWE light bulb was measured with the setup shown in supplement (Fig. S4). It was measured to be 6.98 lm/W and is 32% higher than that of a tungsten light bulb.

Since the white emission occurs only at the spot of the incident focused laser beam, it seems possible to enhance the area of effective lighting of the graphene foam by simultaneous irradiation with several IR laser beams focused at different spots with efficient strong white light sources in mind.

The CIE (CIE 1938) diagram for LIWE of graphene foam in a light bulb shown in Fig. 8c clearly demonstrates yellow shift of the white point, which is very convenient for lighting applications, as warm white light is highly sought after due to human adaptation to natural light sources.

## Summary

The bright white light emission was observed from graphene foam placed in vacuum and irradiated with an IR laser diode. The white light emission was characterized by a very low optical power threshold and rapid build-up and decay times. The LIWE intensity increased nonlinearly with the excitation power and was well scaled according to the power law of multiphoton absorption *I* ∝ *P*^*n*^, where *n* is the order parameter associated with a number of photons involved in the process of multiphoton ionization. The parameter *n* was strongly dependent on laser power density and increased with the decrease of the focus spot area. For the highest pump power density, it was determined to be *n* = 6.1. In the case of irradiation with two laser beams, the total LIWE intensity of graphene foam was a sum of the intensities of the used laser sources. It was observed that the temperature of LIWE graphene foam was relatively low (about 500 °C), which is much less than could be predicted from the black body emission estimation of the lighting temperature. The LIWE process was accompanied by a strong photoelectric effect, with the photoconductance yield increasing exponentially with the applied laser power of similar order as the power dependence of the LIWE intensity. More than one photoelectric process was observed during the laser excitation, showing concurrent increase and decrease of the conductivity. The concurrent process is presumably associated with thermal heating. The efficient electron emission was accompanying the white light emission and its intensity was damped with applied external voltage. It was found that white lighting can be sustained for a long time period. The observed efficient bright white lighting of graphene foam was much more intense than that one observed for graphene ceramic^27^. We suppose that it results from the effective large surface of the porous graphene illuminated by the focused laser beam and a giant nonlinear refractive index n_2_ contributing to the extremely high free electron density. It is demonstrated on a functioning example that such efficient white lighting of graphene foam may be applied in the construction of a new generation of green light bulbs.

## Additional Information

**How to cite this article**: Strek, W. *et al*. Laser induced white lighting of graphene foam. *Sci. Rep.* **7**, 41281; doi: 10.1038/srep41281 (2017).

**Publisher's note:** Springer Nature remains neutral with regard to jurisdictional claims in published maps and institutional affiliations.

## Supplementary Material

Supplementary Information

## References

[b1] Geim A. K. & Novoselov K. S. The rise of graphene. Nat Mater 6, 183–191 (2007). 17330084 10.1038/nmat1849

[b2] Sekiya R., Uemura Y., Murakami H. & Haino T. White-light-emitting edge-functionalized graphene quantum dots. Angew Chemie - Int Ed 53, 5619–5623 (2014). 10.1002/anie.201311248 24711343

[b3] Yu D. & Dai L. Voltage-induced incandescent light emission from large-area graphene films. Appl Phys Lett 96, 2010–2012 (2010).

[b4] Kim Y. D. . Bright visible light emission from graphene. Nat Nanotechnol 10, 1–7 (2015). 26076467 10.1038/nnano.2015.118

[b5] Lim Z. H., Lee A., Zhu Y., Lim K. Y. & Sow C. H. Sustained laser induced incandescence in carbon nanotubes for rapid localized heating. Appl Phys Lett 94, 1–4 (2009).

[b6] Roura P., Costa J., Sardin G., Morante J. R. & Bertran E. Photoluminescence in silicon powder grown by plasma-enhanced chemical-vapor deposition: Evidence of a multistep-multiphoton excitation process. Phys Rev B 50, 18124–18133 (1994). 10.1103/physrevb.50.18124 9976244

[b7] Roura P. . Black-body emission from nanostructured materials. J Lumin 80, 519–522 (1998).

[b8] Roura P. & Costa J. Radiative thermal emission from silicon nanoparticles: a reversed story from quantum to classical theory. Eur J Phys 23, 191 (2002).

[b9] Wang J. & Tanner P. A. Upconversion for white light generation by a single compound. J Am Chem Soc 132, 947–949 (2010). 20025211 10.1021/ja909254u

[b10] Wang J., Hua Hao J. & Tanner P. A. Luminous and tunable white-light upconversion for YAG(Yb_3_Al_5_O_12_) and (Yb,Y)_2_O_3_ nanopowders. Opt Lett 35, 3922 (2010). 21124566 10.1364/OL.35.003922

[b11] Strek W. . White emission of lithium ytterbium tetraphosphate nanocrystals. Opt Express 19, 14083 (2011). 21934770 10.1364/OE.19.014083

[b12] Strek W., Marciniak L., Hreniak D. & Lukowiak A. Anti-Stokes bright yellowish emission of NdAlO_3_ nanocrystals. J Appl Phys 111, doi: 10.1063/1.3674272 (2012).

[b13] Marciniak L. . Upconversion emission of LiNdP_4_O_12_ and KNdP_4_O_12_ crystals. J Lumin 133, 57–60 (2013).

[b14] Chen X. . Nd_2_O_3_/Au nanocomposites: Upconversion Broadband Emission and Enhancement under Near-infrared Light Excitation. J Mater Chem C 5857–5863 (2014).

[b15] Strek W. . Broadband anti-Stokes white emission of Sr2CeO4 nanocrystals induced by laser irradiation, Phys. Chem. Chem. Phys. 18, 27921–27927, doi: 10.1039/c6cp04904d (2016). 27722306

[b16] Wang J. . Photon energy upconversion through thermal radiation with the power efficiency reaching 16%. Nat Commun 5, 5669 (2014). 25430519 10.1038/ncomms6669

[b17] Singh K. . Light-into-heat conversion in La_2_O_3_:Er(^3+^)-Yb^3+^ phosphor: an incandescent emission. Opt Lett 37, 776–8 (2012). 22378390 10.1364/OL.37.000776

[b18] Zhu Y. . Broad White Light and Infrared Emission Bands in YVO4:Yb^3+^, Ln^3+^ (Ln^3+^ = Er^3+^, Tm^3+^, or Ho^3+^). Appl Phys Express 5, 092701 (2012).

[b19] Bilir G. . Broadband Visible Light Emission From Nominally Undoped and Cr^3+^ Doped Garnet Nanopowders. IEEE Photonics J 6, 1–11 (2014).

[b20] Bilir G., Ozen G. & Di Bartolo B. Peculiar effects accompanying the production of white light by IR excited nanoparticles. Opt Spectrosc 118, 131–134 (2015).

[b21] Bilir G. & Di Bartolo B. Production of bright, wideband white light from Y_2_O_3_ nano-powders induced by laser diode emission. Opt Mater (Amst) 36, 1357–1360 (2014).

[b22] Erdem M., Eryurek G. & Di Bartolo B. White light emission from sol-gel derived γ-Y_2_Si_2_O_7_ nanoparticles. J Alloys Compd 639, 483–487 (2015).

[b23] Cesaria M., Collins J. & Di Bartolo B. On the efficient warm white-light emission from nano-sized Y_2_O_3_. J Lumin 169, 574–580 (2016).

[b24] Lim Z. H., Lee A., Lim K. Y. Y., Zhu Y. & Sow C. H. Systematic investigation of sustained laser-induced incandescence in carbon nanotubes. J Appl Phys 107, 1–8 (2010).

[b25] Zeng H., Yang C., Dai J. & Cui X. Light-induced incandescence of single-walled carbon nanotubes. J Phys Chem C 112, 4172–4175 (2008).

[b26] Schulz C. . Laser-induced incandescence: Recent trends and current questions. Appl Phys B Lasers Opt 83, 333–354 (2006). 10.1007/s00340-022-07769-z PMC8921179 35308124

[b27] Strek W. . Laser-induced white-light emission from graphene ceramics–opening a band gap in graphene. Light Sci Appl 4, e237 (2015).

[b28] Keldysh L. V. Ionization in the Field of a Strong Electromagnetic Wave. Soviet Physics JETP 20, 1307 (1965).

[b29] Yablonovich E. & Bloembergen N. Avalanche Ionization and the Limiting diameter of Filaments Induced by Light Pulses in Transparent Media. Phys. Rev. Lett. 29, 907 (1972).

[b30] Richardson O. W. Electron emission from metals as a function of temperature. Phys. Rev. 23, 153–155, (1924).

[b31] Zhang T., Chang H., Wu Y. . Macroscopic and direct light propulsion of bulk graphene materia, Nature Photonics 9 471 (2015).

[b32] Zhang H., Virally S., Bao Q. . Optics Letters 37, 18561858 (2012). 10.1364/OL.37.001856 22660052

[b33] Raman R. K. . Direct Observation of Optically Induced Transient Structures in Graphite Using Ultrafast Electron Crystallography, Phys. Rev. Lett. 101, 077401 (2008). 18764578 10.1103/PhysRevLett.101.077401

