# Supplementary material for: Laser induced white lighting of graphene foam
Source: Sci Rep. 2017 Jan 23;7:41281. doi: 10.1038/srep41281 (PMC5256029; doi:10.1038/srep41281)
Supplement: Supplementary Information [file srep41281-s1.doc]

**Supplementary information for**

**Laser induced white lighting of graphene foam**

Wieslaw Strek, Robert Tomala*, Mikolaj Lukaszewicz, Bartlomiej Cichy, Yuriy Gerasimchuk,

Pawel Gluchowski, Lukasz Marciniak, Artur Bednarkiewicz, Dariusz Hreniak

Institute of Low Temperatures and Structural Research,

Polish Academy of Science, 50-422 Wroclaw, Poland

*corresponding author : R. Tomala, Polish Academy of Sciences, Institute of Low Temperature and Structure Research, Okolna 2, 50-422 Wroclaw, Poland

E-mail: r.tomala@int.pan.wroc.pl

***S1. Materials preparation and morphology***

The graphene foam^1^ was synthesized according to the following procedure. In the first step, 0.2g of highly oxidized graphite oxide (GO) (obtained by modified Brodie method, described by earlier in^2^) was added to 20 cm^3^ of deionized water contained in a 40 cm^3^ polypropylene vial. The vial was then placed in a high power ultrasonic dispergator (22 kHz, 900W) for 30 min. After the sonification and obtaining of a stable suspension, 0,088g of resorcinol (98%, SAFC, MO, USA) and 0,068 ml of formalin (35% water solution of formaldehyde, Sigma-Aldrich, Germany) were added to the solution and placed on the magnetic stirrer for intensive stirring. Simultaneously, solution of 0,425g of Na_2_CO_3_ (>99%, Alfa-Aesar, Germany) in 10 ml of deionized water was prepared. After 1 hour of stirring, 10 mm^3^ of the sodium carbonate solution was added to the GO suspension, followed by intense stirring for the next 5 hours. Afterwards, the vial was closed and placed in a laboratory dryer in 85°C for gelation (which takes 3-4 days). After gelation, the vial was opened and placed in a low temperature vacuum dryer for a 2 days. Finally, pyrolysis of obtained probe in a tube furnace in 1050°C in nitrogen atmosphere was performed for 1 hour.

Morphology of the as- prepared graphene foam was analyzed by the SEM microscopy. Representative image of the sample is shown in Fig. S1a. One may observe the well-developed macroporous foam structure of the samples characterized by large pores and sharp edges formed mostly by single graphene sheets. The average size of the foam pore was estimated to *c.a.* 5 µm.


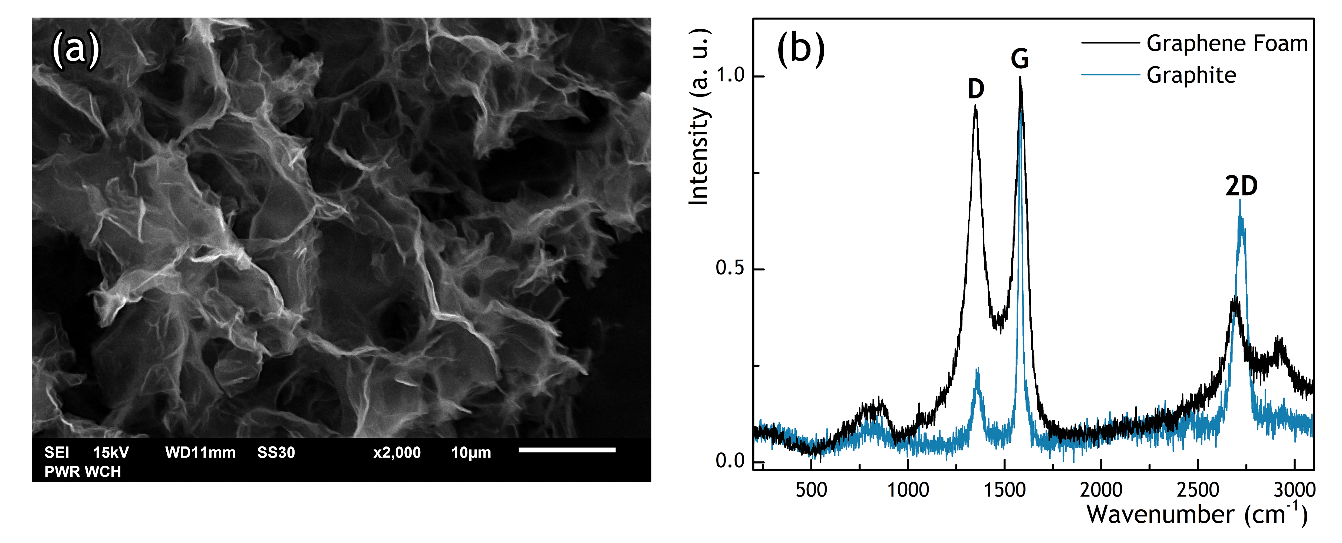


Fig. S1 (a) Representative SEM image of the foamed graphene and its surface morphology. The image was taken on as-prepared samples without further modifications. (b) Raman spectrum of the graphene foam. The Raman spectrum of graphite is added for comparison.

Structure of the as-prepared graphene foam and pristine graphene content was investigated according to the vibrational properties of the sample. The Raman spectra of the FG along with representative spectra of pristine graphene and graphite were shown in Fig. 2. Three modes located around 1350 cm^-1^, 1580 cm^-1^ and 2700 cm^-1^ characteristic for different allotropes of carbon were analyzed. The D mode arises from a breathing mode of κ-point photons of A1g symmetry (ca. 1350 cm^-1^). The G band is associated to the doubly degenerated E2g phonon mode at the Brillouin zone center. This band (near 1580 cm^-1^) arises due to the in-plane vibration of the sp^2^ carbon atoms. G-band arises from the stretching of the C- C bond in graphitic materials to all sp^2^ carbon systems. The G-band is highly sensitive to strain effects in sp^2^ system, and thus can be used to probe modification on the flat surface of graphene. The G band peak may be accompanied by a shoulder peak at 1620 cm^-1^ (D' band)^3^, which is associated with finite-size graphite crystals, but in case of graphene and graphene foam it was not observed that confirms only graphene phase. The 2D band (at 2700 cm^-1^) is at almost double the frequency of the D band and originates from second order Raman scattering process. The 2D band peak at 2700 cm^-1^ is often used to confirm the presence of graphene and number of its layers^4^ and it originates from a double resonance process that links phonons to the electronic band structure. The side band at 2900 cm^-1^ may be result of reducing graphene in nitrogen atmosphere^5^.

***S2. Pressure dependence of LIWE***

The sample pumped with an intense laser beam enhances its temperature due to the absorption of light and the heat flowing to a colder environment. The energy balance equation is expressed by^6^

*P_ab_(I_L_) = P_em_(T) + C_p_*
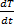
 *(1)*

where *C_p_* is the heat capacity and *I_L_* is the laser intensity. *P_ab_*
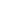

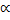
 *AI*_L_ is the power absorbed from laser irradiation and *P_em_* is the power dissipated by emitting particles. In vacuum, the dissipation of excitation energy in illuminated sample is reduced almost to zero and all absorbed energy is transferred into the emission of white light, enhancing the temperature of the sample. The influence of the atmosphere pressure on LIWE intensity is presented in Fig. S1.


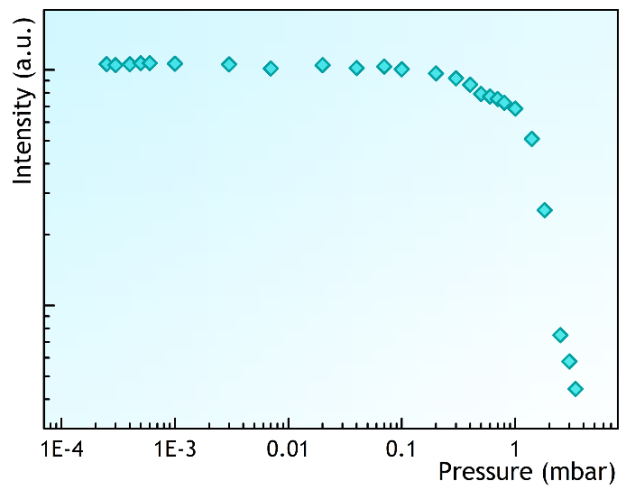


Fig. S2. The influence of the atmosphere pressure on the emission intensity of the graphene foam irradiated with a 975 nm CW laser beam.

***S3. Temperature of LIWE graphene foam***

The temperature of the graphene foam LIWE was determined using the nanothermometry technique with LiLaP_4_O_12_:Er^3+^,Yb^3^*^+^* nanocrystals as thermal sensors placed at the surface of graphene foam^7^. The temperature has been calculated from the ratio of ^2^H_11/2_→^4^I_15/2_ and ^4^S_3/2_→^4^I_15/2_ Er^3+^ transitions. The spectra of LIWE with thermal sensors are presented on Fig. S3. In the inset of this figure the impact of excitation power density on the temperature is shown. The temperature increases linearly with the increase of the excitation power density and under 4500 W/cm^2^ it was estimated to be about 500°C.


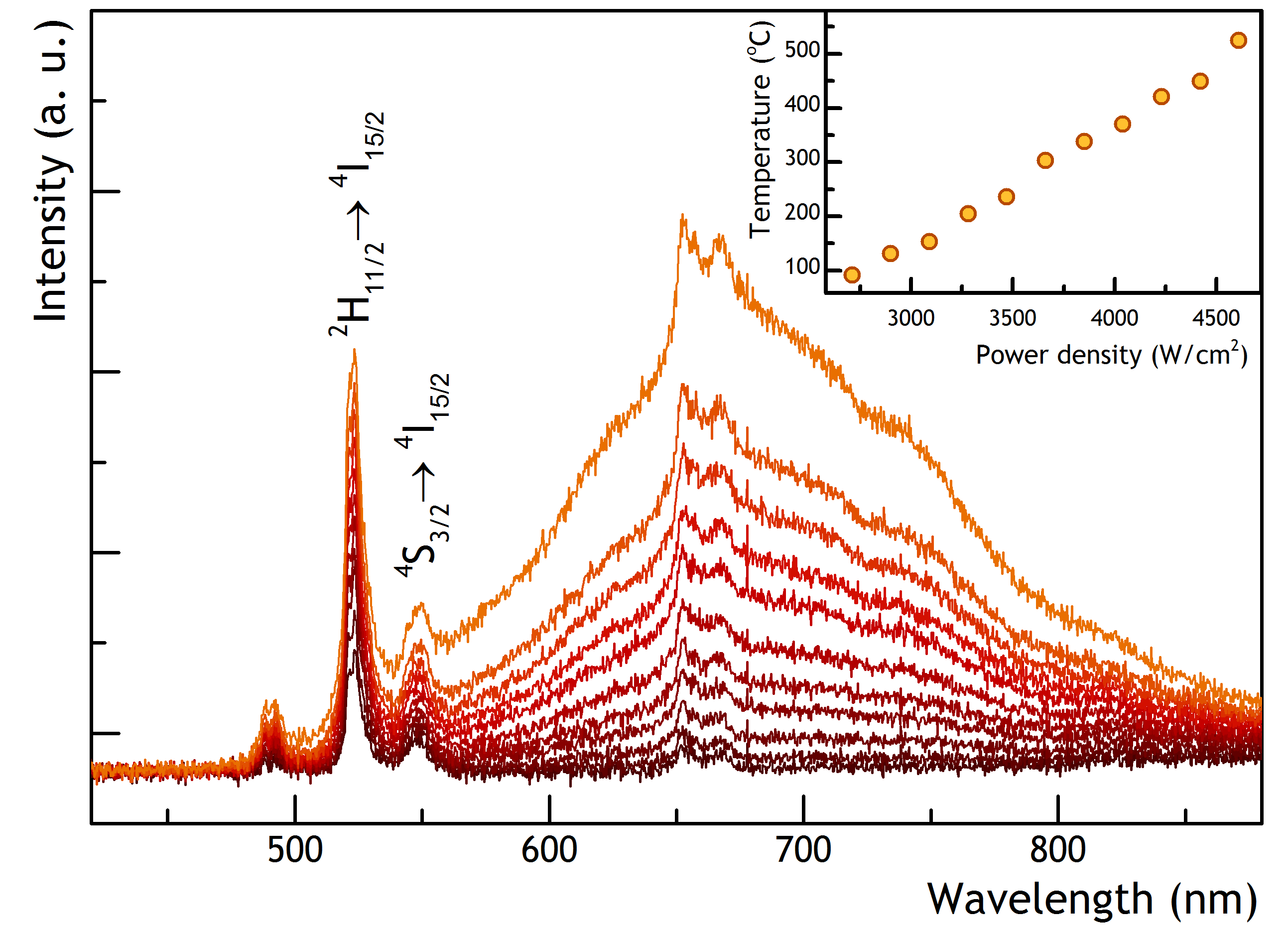


Fig. S3. The evaluation of spectra of graphene foam with LiLaP_4_O_12_:Er^3+^,Yb^3^*^+^* nanocrystals as thermal sensors with changing 975 nm LD power density. The calculated temperatures are presented in the inset.

***S4. Power efficiency measurement***

The power efficiency experiment of the graphene foam irradiated by a 975 nm laser diode was performed using Gigahertz-Optik ISD-21-BTS256-LED spectroradiometer with a 21 cm diameter integration sphere. This equipment is suitable for measuring a light source with a diameter of up to 63.5 mm. The scheme of the setup is shown in Fig. S4. The measured value of the luminous flux of graphene foam was 3.2 lm upon 1.4 W excitation, giving the luminous efficacy of 2.28 lm/W. For comparison, the luminous efficiency of the 15 W tungsten lamp was determined to be 5.2 lm/W. The measurement of the luminous flux inside the integration sphere is not beneficial for such a light source, as the most of light is emitted in the direction reverse to the excitation source and only a part of the light is registered. It is exactly for this reason that for the calculations we have taken into account the influence of the angle of the detector position on the emission intensity. An overall luminous efficiency was determined to be 6.90 lm/W. Meaning that the effective efficiency was 32% larger in comparison to the tungsten bulb.

The power efficiency measurements of LIWE light sources based on oxide lanthanides were reported by Wang and Tanner^8^ on Tm_2_O_3_ to be 17% more efficient than the tungsten bulb and by Bilir and Di Bartolo^9^ on Y_2_O_3_ to be 38% more efficient than the tungsten bulb.


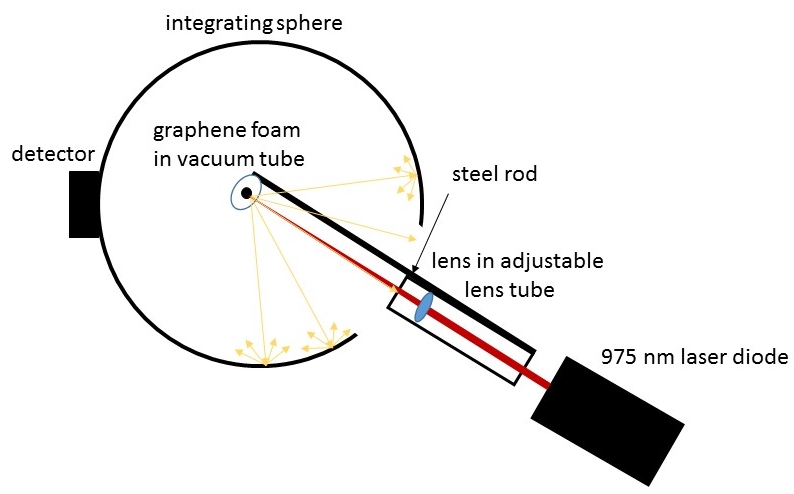


Fig S4. Schematic of the experimental setup of the luminous flux measurement.

**References**

1. Worsley MA, Pauzauskie PJ, Olson TY, Bienier J, Satcher JH, Baumann TF, Synthesis of Graphene Aerogel with High Electrical Conductivity, J. Am. Chem. Soc.,2010; 132 (40), 14067–14069.
2. Kędziora A, Gerasymchuk Y, Sroka E, Bugla-Płoskońskam G, Doroszkiewiczm W, Rybak Z, Hreniak D, Stręk W, Use of the Materials Based on Partially Reduced Graphene-Oxide with Silver Nanoparticle as Bacteriostatic and Bactericidal Agent, Polim. Med., 2016; 43, 129–134.
3. Ferrari AC, Meyer JC, Scardaci V, Casiraghi C, Lazzeri M, Mauri F, Poscanec S, Jiang D, Novoselov KS, Roth S, Geim AK, Raman Spectrum of Graphene and Graphene Layers, Physical Review Letters, Phys. Rev. Lett., 2006; 97, 187401.
4. Wang YY, Ni ZH, Yu T, Shen ZX, Wang HM, Wu YH, Chen W, Wee ATS, Raman Studies of Monolayer Graphene: The Substrate Effect, J. Phys Chem. C, 2008; 112, 10637–10640.
5. Xue Y, Chen H, Qu J, Dai L, Nitrogen-doped graphene by ball-milling graphite with melamine for energy conversion and storage, 2D Materials, 2015; 2, 044001.
6. Roura P, Costa J. Radiative thermal emission from silicon nanoparticles: a reversed story from quantum to classical theory. *Eur J Phys* 2002; **23**: 191.
7. Strek W, Cichy B, Radosinski L, Gluchowski P, Marciniak L, Lukaszewicz M *et al.* Laser-induced white-light emission from graphene ceramics–opening a band gap in graphene. *Light Sci Appl* 2015; **4**: e237.
8. Wang J., Tanner P.A., Upconversion for white light generation by a single compound, J. Am. Chem. Soc. 132 (2010) 947–949
9. Bilir G., Di Bartolo B., Production of bright, wideband white light from Y_2_O_3_ nano-powders induced by laser diode emission, Opt. Mater. 2014; 36; 1357-1360
